# Identification of hub glutamine metabolism-associated genes and immune characteristics in pre-eclampsia

**DOI:** 10.1371/journal.pone.0303471

**Published:** 2024-05-08

**Authors:** Yan Mao, Xinye Li, Rui Ren, Yue Yuan, Li Wang, Xuehong Zhang

**Affiliations:** 1 First School of Clinical Medicine, Lanzhou University, Lanzhou, Gansu, China; 2 Department of Gynecology and Obstetrics, Gansu Provincial Hospital, Lanzhou, Gansu, China; 3 Department of Gynecology and Obstetrics, General Hospital of Lanzhou Petrochemical Corporation, Lanzhou, Gansu, China; 4 Gansu Key Laboratory for Reproductive Medicine and Embryology, The First Hospital of Lanzhou University, Lanzhou, Gansu, China; The First Affiliated Hospital of Nanjing Medical University, CHINA

## Abstract

**Objective:**

Preeclampsia (PE) is a severe complication of unclear pathogenesis associated with pregnancy. This research aimed to elucidate the properties of immune cell infiltration and potential biomarkers of PE based on bioinformatics analysis.

**Materials and methods:**

Two PE datasets were imported from the Gene ExpressioOmnibus (GEO) and screened to identify differentially expressed genes (DEGs). Significant module genes were identified by weighted gene co-expression network analysis (WGCNA). DEGs that interacted with key module genes (GLu-DEGs) were analyzed further by Kyoto Encyclopedia of Genes and Genomes (KEGG) and Gene Ontology (GO) analyses. The diagnostic value of the genes was assessed using receiver operating characteristic (ROC) curves and protein-protein interaction (PPI) networks were constructed using GeneMANIA, and GSVA analysis was performed using the MSigDB database. Immune cell infiltration was analyzed using the TISIDB database, and StarBase and Cytoscape were used to construct an RBP-mRNA network. The identified hub genes were validated in two independent datasets. For further confirmation, placental tissue from healthy pregnant women and women with PE were collected and analyzed using both RT-qPCR and immunohistochemistry.

**Results:**

A total of seven GLu-DEGs were obtained and were found to be involved in pathways associated with the transport of sulfur compounds, PPAR signaling, and energy metabolism, shown by GO and KEGG analyses. GSVA indicated significant increases in adipocytokine signaling. Furthermore, single-sample Gene Set Enrichment Analysis (ssGSEA) indicated that the levels of activated B cells and T follicular helper cells were significantly increased in the PE group and were negatively correlated with GLu-DEGs, suggesting their potential importance.

**Conclusion:**

In summary, the results showed a correlation between glutamine metabolism and immune cells, providing new insights into the understandingPE pathogenesis and furnishing evidence for future advances in the treatment of this disease.

## Introduction

Preeclampsia (PE) is a progressive multisystem disease that is unique to pregnancy and significantly threatens the life and health of mothers and children. The average global incidence of PE is 3‒8% [1]. The PE-associated pathophysiological changes develop in early pregnancy; however, hypertension and proteinuria are observed after 20 weeks with or without progressive multi-organ damage. Currently, clinical therapy is limited to symptomatic treatment, and there are no effective preventive measures to improve the health of the mother and child. Emergency cesarean sections are often performed, resulting in serious iatrogenic preterm birth and complications associated with cesarean sections [2]. Therefore, it is important to identify markers of PE that will allow its early identification, thus reducing the maternal mortality rate and improving the neonatal survival rate.

Immunological factors are thought to be significantly associated with the etiology and pathogenesis of PE. Immune homeostasis is essential for the maintenance of pregnancy, and studies have indicated that abnormal activation of the immune system during pregnancy might be significantly associated with PE development [3], such as the activation of monocytes and neutrophils, abnormal cytokine production, and imbalances between regulatory T cells and helper T cells [4].

Glutamine (GLU) is the most abundant amino acid in the circulation and is rapidly utilized by tumor cells. It is frequently utilized as a citrate source for lipid production in reductive carboxylation or for maintaining TCA flux in cellular aerobic glycolysis. Furthermore, glutaminolysis assists the survival of proliferating cells by reducing oxidative stress and maintaining mitochondrial membrane integrity. It also contributes to the energy supplies of immune and tumor cells. However, inflammatory immune cells such as macrophages that act against the tumors do not utilize GLU metabolism. In comparison with naïve macrophages, M2 macrophages are dependent on GLU, whereas inhibition of GLU metabolism induces the pro-inflammatory M1 macrophage phenotype [5]. Thus, GLU metabolism is a possible target for the phenotypic conversion of tumor-associated macrophages (TAMs) from the M2 to the M1 phenotype, which increases the anti-tumor responses of immune-mediated inflammation. Additionally, GLU metabolism is associated with Th1 cell differentiation and effector T cell activation [6]. These data indicate that targeting of GLU metabolism can remodel the tumor microenvironment (TME) and enhance the efficacy of immunotherapy. It has been reported that suppression of GLU metabolism substantially elevates the efficacy of anti-PD-1 anti-tumor treatment and the cytotoxic effects of effector T cells due to the metabolic reprogramming [7]. Therefore, targeting appropriate metabolic pathways can stimulate inflammatory immunity and block tumor metabolism, which are crucial for improving immunotherapy, and GLU metabolism is among those potential targets.

In current years, bioinformatics methods have been extensively used for the analysis of microarray and high-throughput data, including the identification of differentially expressed genes (DEGs). These methods are also useful for analyzing the underlying pathways of various human diseases. Since metabolic abnormalities and immune cell infiltration are important in the etiology of PE, the present study used the comprehensive genomic assessment of public databases to find new early diagnostic biomarkers and therapeutic targets by identifying key GLU-DEG modules (genes related to GLU metabolism), pathways, and immune infiltrates to provide ideas for promoting further research in this field.

## Materials and methods

### Data sources and preprocessing

This investigation used publicly available data, mainly imported from the Gene Expression Omnibus (GEO, https://www.ncbi.nlm.nih. gov/geo/). The flowchart of this study can be found in S1 Fig. Genome-wide expression data related to PE were downloaded using the R package ‘GEOquery’ from the GEO database. The GSE186257 dataset comprised 26 PE patients and 18 controls, while the GSE96984 dataset included 3 PE patients and 4 controls, GSE24129 contained 8 PE patients and 8 controls, and GSE35574 included 19 PE patients and 40 controls. Non-biological technical biases inducing batch effects were corrected with the help of the ComBat protocol of the “sva” R package [8]. Moreover, the correction degree was assessed using principal component analysis (PCA). This investigation honored each database’s data access policies.

A total of 134 GLU metabolism-linked genes were acquired from the Msigdb database (http://www.gseamsigdb.org/gsea/msigdb/index.jsp) [9,10].

### PE-associated DEGs

The DEGs between control (n = 22) and PE (n = 29) samples were identified using the “limma" package (version 3.50.0) in R [11]. The parameters were set as |log2 fold change| > 0.5 and adjusted *p < 0*.*05*. A heatmap was established using the “pheatmap” package in R with a complete linkage clustering method and Euclidean distance.

### Gene Set Variation Analysis (GSVA)

The GSVA is a non-parametric, unsupervised gene enrichment method that utilizes gene expression profiles to identify the correlation of genetic features with biological pathways. To elucidate the difference in the biological role between the control and PE cohorts, GSVA was carried out with “c2.cp.kegg.v7.5.1.symbols” using the R “GSVA" (v 1.42.0) package. The “pheatmap" (v 1.0.12) package in R was used for visualization of the results. Furthermore, 50 HALLMARK gene sets were imported from the MSigDB database (http://software.broadinstitute.org/gsea/msigdb) and used as a reference. The ssGSEA function in GSVA was utilized to assess the GSVA score of each gene set in various samples. These scores were then compared between the control and PE groups using the "limma" package.

### Weighted Gene Co‑expression Network Analysis (WGCNA) and assessment of significant modules

The WGCNA algorithm in the R WGCNA package (v 1.70–3) was employed for the construction of co-expression networks [12]. Similarities in gene expressed were analyzed using Pearson correlation coefficients and were weighted using a power function to produce a scale-free network. The ‘PickSoftThreshold’ package in R was used to establish a weighted adjacency matrix by increasing the co-expression similarity to a power β = 22. In terms of co-expression, a gene module represents a densely interconnected gene cluster. WGCNA utilizes hierarchical clustering to assess gene modules, which are indicated by different colors. Furthermore, various modules were identified with the help of the dynamic tree-cut method. Cluster analysis was utilized for module selection, during which the adjacency matrix (a topology similarity measure) was transformed into a topology overlay matrix (TOM). To identify the link between modules and GLU, the relationship of the module eigengene (ME, the module’s 1^st^ principal element that depicts its overall expression level) to GLU was elucidated by Pearson’s correlations. Modules showing significant links with GLU were identified, and heatmaps of the topological overlap in the gene network were generated to observe co-expression module structure. The hierarchical clustering dendrogram of the eigengenes and the corresponding eigengene network heatmap were used to summarize the links between modules. The GLU-related DEGs were acquired by intersecting DEGs and genes from the GLU-related module.

### Kyoto Encyclopedia of Genes and Genomes (KEGG) pathway enrichment and Gene Ontology (GO) analyses

The GO categories are molecular function (MF), biological process (BP), and cellular component (CC). KEGG is a bioinformatics tool for identifying pathways in which genes are significantly enriched [13]. The KEGG enrichment and GO analyses were performed using the “clusterProfiler" (v 4.2.2) package in R (p-value < 0.05) using the GLU-related DEGs [14].

### GeneMANIA

The GeneMANIA website (http://genemania.org) was used to construct protein-protein interaction (PPI) networks. GeneMANIA can also predict the link between hub genes and functionally similar genes, such as protein-DNA interaction or PPI, biochemical and physiological reactions, pathways, co-expression, and co-localization [15].

### Receiver Operating Characteristic (ROC) curve

The ROC curve is a plot with test sensitivity on the y-coordinate *vs*. its 1-specificity or false positive rate (FPR) on the x-coordinate. ROC curves can effectively elucidate the efficiency of diagnostic assays. The most common metric acquired from the ROC plot of sensitivity *vs*. 1–1-specificity is the area under the curve (AUC). Here, “pROC” [PMID: 21414208] in R was utilized to generate ROC curves to elucidate the AUC values for identifying signature genes and elucidating their diagnostic potential [16]. It was assessed on a 0.5 (a “coin flip”) to 1 (perfect discrimination) scale. Generally, an AUC of > 0.9 = outstanding, 0.8–0.9 = excellent, 0.6–0.8 = acceptable, and 0.5 = no discrimination.

### Immune infiltration analysis

The ssGSEA is a variation of the GSEA algorithm that, instead of assessing enrichment scores for various groups (disease *vs*. control) and gene sets (i.e., pathways), provides a score for each gene set pair and sample [17]. Each ssGSEA enrichment score indicates how many genes in a specific set within a sample are down or up-regulated.

Based on the 28 types of immune cells acquired from the Tumor and Immune System Interactions Database (TISIDB) (http://cis.hku.hk/TISIDB/index.php) [18], including eosinophils, regulatory T cells, activated, immature, and memory B cells, natural killer (NK) cells, gamma delta T cells, CD56 bright and dim NK cells, Type 1, 17, and 2 T helper cells, myeloid-derived suppressor cells, central memory, activated, and effector memory CD8 T and CD4 T cells, T follicular helper cells, macrophages, NK T cells, activated, plasmacytoids, and immature dendritic cells, monocytes, neutrophils, and mast cells, the relative enrichment score of each type of immune cell was determined from each sample’s gene expression profile. Variations in the immune cell infiltration levels between PE and control samples were evaluated using the R package "ggplot2" (v 3.3.6) [19].

### Construction of the RBP–mRNA network

StarBase (https://starbase.sysu.edu.cn/tutorialAPI.php#RBPTarget) is a widely utilized open-source platform for assessing the interactions of ncRNA *via* degradome-seq, RNA-RNA interactome, and CLIP-seq data. StarBase was utilized to elucidate the link between the expression of mRNA and RNA-binding proteins (RBPs). A clusterNum ≥ 5, *p-value < 0*.*05*, and clipExpNum ≥ 5 were used as the cutoff parameters for determining the key mRNA-RBP pairs in PE. Furthermore, using Cytoscape, the RBP-mRNA network was generated.

### Sample collection

Samples were acquired from women who were registered in the Department of Obstetrics and Gynecology of the Gansu Provincial Hospital for cesarean section from April 1 to September 30, 2023. This research was authorized by the Medical Ethics Committee of the Gansu Provincial Hospital (approval no. 2023–039). Participants were first informed about the research, and then provided written consent. There were two cohorts, namely, health control (HC) and PE. The placental tissue was sampled immediately after the surgery, with each sample divided into two portions: one immersed in RNAsolid tissue RNA stabilization reagent (Servicebio,China) and stored at -80°C until utilized for RT-PCR; the other portion was fixed in polyformalin tissue fixative (Servicebio,China), embedded in paraffin, and utilized for immunohistochemistry.

### Immunohistochemistry (IHC)

The paraffin sections were de-waxed and rehydrated,and antigen retrieval was carried out by heating in a microwave oven in citrate buffer. Endogenous peroxidase activity was quenched with 3% hydrogen peroxide, and non-specific binding was blocked with 5% goat serum (Solarbio,China). The slides were then incubated overnight at 4°C with primary antibodies targeting ACSS1 (Proteintech,China), SLC27A2 (Thermofisher,USA), ADAMTS19 (Abcam,USA) and LGR5 (Theroma,USA). Following PBS washing steps, secondary antibodies were incubated, and subsequent incubation with freshly prepared DAB (Solarbio,China) working solution ensued. Counterstaining was performed using hematoxylin, and examined under a microscope.

### RT-qPCR

Total RNA was extracted from the placental tissue after cesarean section using TRIzol (TaKaRa, Japan). The cDNA was synthesized per the kit’s protocol (TaKaRa). GAPDH was used as the internal reference, and relative expression of genes was calculated using the 2^−ΔΔCT^ method. qRT-PCR was repeated three times for each sample. The primer sequences used are shown below:

GAPDH

Forward: 5′- TCAAGAAGGTGGTGAAGCAGG-3′,

Reverse: 5′- AAAGGTGGAGGAGTGGGTGTC-3′.

ASSC1

Forward: 5′- TGGCTCACAGGACAGACAACAAG-3′,

Reverse: 5′- GGCGGCATAGAGCAGGTAGC-3’.

LGR5

Forward: 5′- CTCGGTGTGCTCCTGTCCTTG-3′,

Reverse: 5′- GAGGTGAAGACGCTGAGGTTGG-3’.

SLC27A2

Forward: 5′-GCTCCTGGTGAACCTCTGCTG-3′,

Reverse: 5′- AAGGCTTGTGTGGCGTCTGG-3’.

ADAMTS19

Forward: 5′- GAATGGACCCCTTGTTCACGA-3′,

Reverse: 5′-TACATCTAACGGTCCGATGACG-3’.

### Statistical analysis

Statistical assessments were conducted using R software v4.1.2. Associations between parameters were assessed by Spearman correlations. Differences between two groups were analyzed using Wilcoxon tests, while differences between multiple groups were assessed by Kruskal–Wallis tests. P-values < 0.05 were considered statistically significant.

## Results

### Weighted gene co-expression network construction and module identification

**Fig 1:**
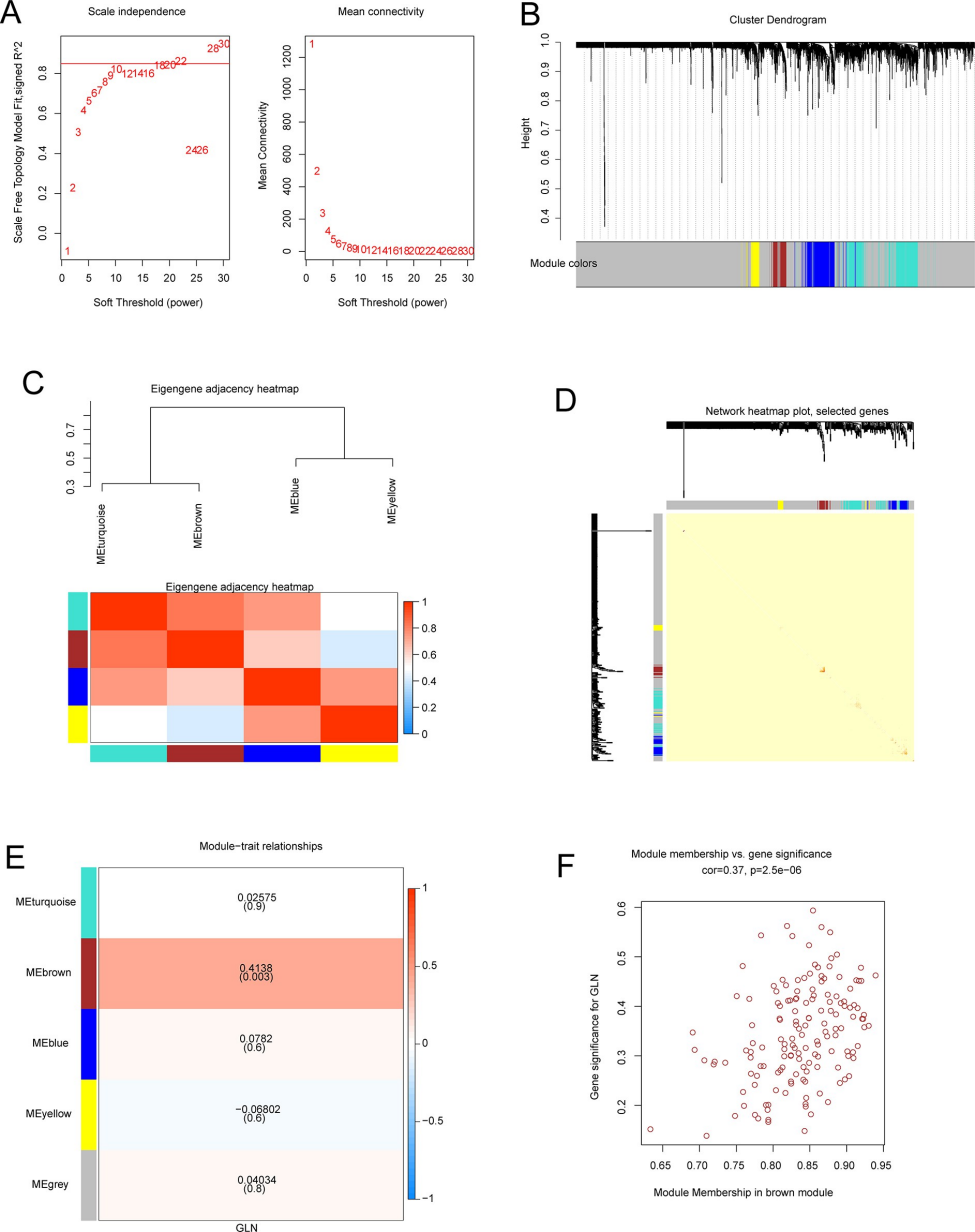
Establishing the WGCNA co-expression network. (A) Scale-free topological fit index (R2) and soft threshold β = 22. (B) Gene expression network analysis in PE determined specific co-expression data modules. (C) Associations between modules. Top: hierarchical clustering of module eigengenes summarizing the modules identified *via* the clustering test. Branches of the dendrogram (the meta-modules) group positively correlated eigengenes. Bottom: Heatmap plot of the adjacencies in the eigengene network. In the heatmap, each column and row indicates one module eigengene (color labeled). In the heatmap, blue = low adjacency and red = high adjacency. Red colored squares observed along the diagonal represent the meta-modules. (D) Heatmap of topological overlap in the gene network. In the heatmap, each column and row indicates a gene; progressively darker red color indicates higher topological overlap while light color indicates low topological overlap. The darker squares along the diagonal represent modules. The module assignment and gene dendrogram are indicated along the top and left. (E) Association of GLU with consensus module eigengenes. In the table, each column prepresents a sample or trait, and each row represents a consensus module. Numbers in the table indicate the link between the traits and the corresponding module eigengenes. The p-values are provided in parentheses below the correlations. Based on the color legend, the table is color-coded by correlation. (F) Correlation between gene significance (GS) and module membership (MM) for GLU of genes of the brown module. ‘Cor’ depicts the absolute correlation coefficient between MM and GS.

The identification of GLU-related gene sets was undertaken using WGCNA. The scale independence and mean connectivity analysis indicated that the average degree of connectivity was close to 0, and scale independence was > 0.85 at the weighted value = 22 (Fig 1A). Altogether, 4 co-expressed modules were determined, and a gray module was assigned for uncorrelated genes, which was subsequently ignored (Fig 1B). To investigate the link between the modules and assess their correlation, the MEs were correlated. Fig 1C illustrates the eigengene network *via* a dendrogram and a heatmap plot. The topological overlap in the gene network was represented in a heatmap plot (Fig 1D). To understand the physiological importance of the modules, 4 MEs were correlated with GLU, and the most significant associations were assessed. The module-trait correlation heatmap (Fig 1E) indicates that the brown module gene cluster (n = 153) had the strongest positive association with GLU (r = 0.41, *p < 0*.*05*). Therefore, the brown module was used in subsequent analyses as it indicated GLU more accurately. Fig 1F indicates the scatterplots of gene significance (GS) for the trait of GLU *vs*. module membership (MM) in the brown module. MM and GS for GLU had markedly positive associations (cor = 0.37, *p < 0*.*05*), suggesting that the brown module’s central (most important) elements were highly correlated with the GLU trait.

### DEG identification

**Fig 2:**
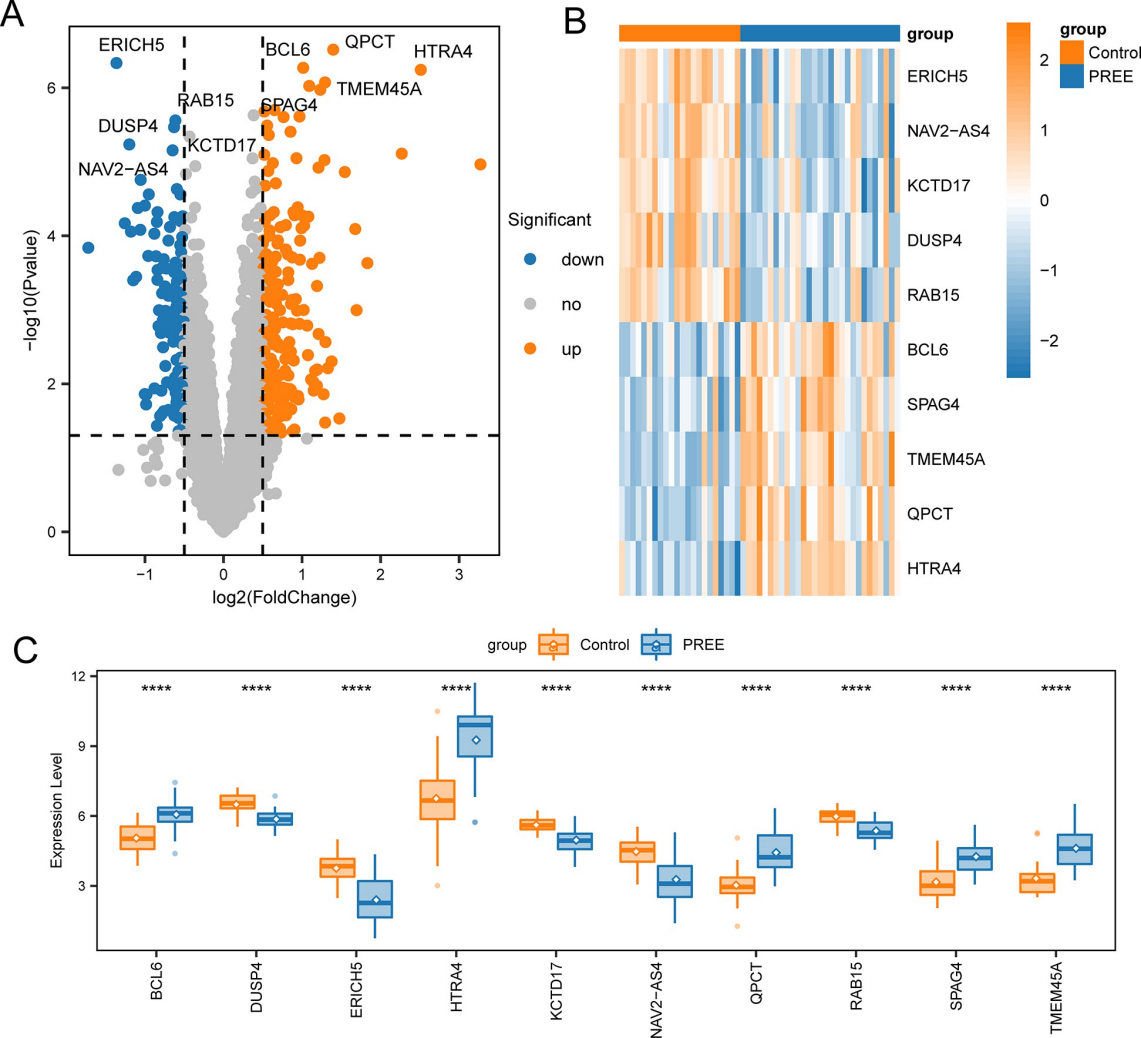
DEG identification. (A) Volcano plot of DEGs between the control and PE samples. Gray dots indicate downregulated, upregulated, and insignificant gene expression levels. (B) Heatmap of the top 5 downregulated and upregulated DEGs, respectively. (C) Differences in the expression of the top 10 DEGs between the control and PE cohorts, determined by Wilcoxon tests. Asterisks indicate p-values (*p < 0.*05*, **p < 0.*01*, ***p < 0.*001*, ****p < 0.*0001*).

The comparison of the PE samples and controls identified 356 significantt DEGs between the two cohorts (adjusted *p-value* < 0.05, |Log2-fold change| > 0.5). In PE specimens, 128 and 228 genes were downregulated and upregulated, respectively. A volcano plot was employed for visualization of the DEGs (Fig 2A). The top five upregulated (BCL6, SPAG4, TMEM45A, QPCT, and HTRA4) and downregulated DEGs (ERICH5, NAV2-AS4, KCTD17, DUSP4, and RAB15) are shown in the heatmap (Fig 2B). Wilcoxon tests showed that the expression of these top 10 genes differed significantly between the two cohorts (*p < 0*.*05*, Fig 2C).

A total of 7 GLU-related DEGs were identifed in the intersection between DEGs and GLU-related module genes; these were considered hub genes.

### GSVA

**Fig 3:**
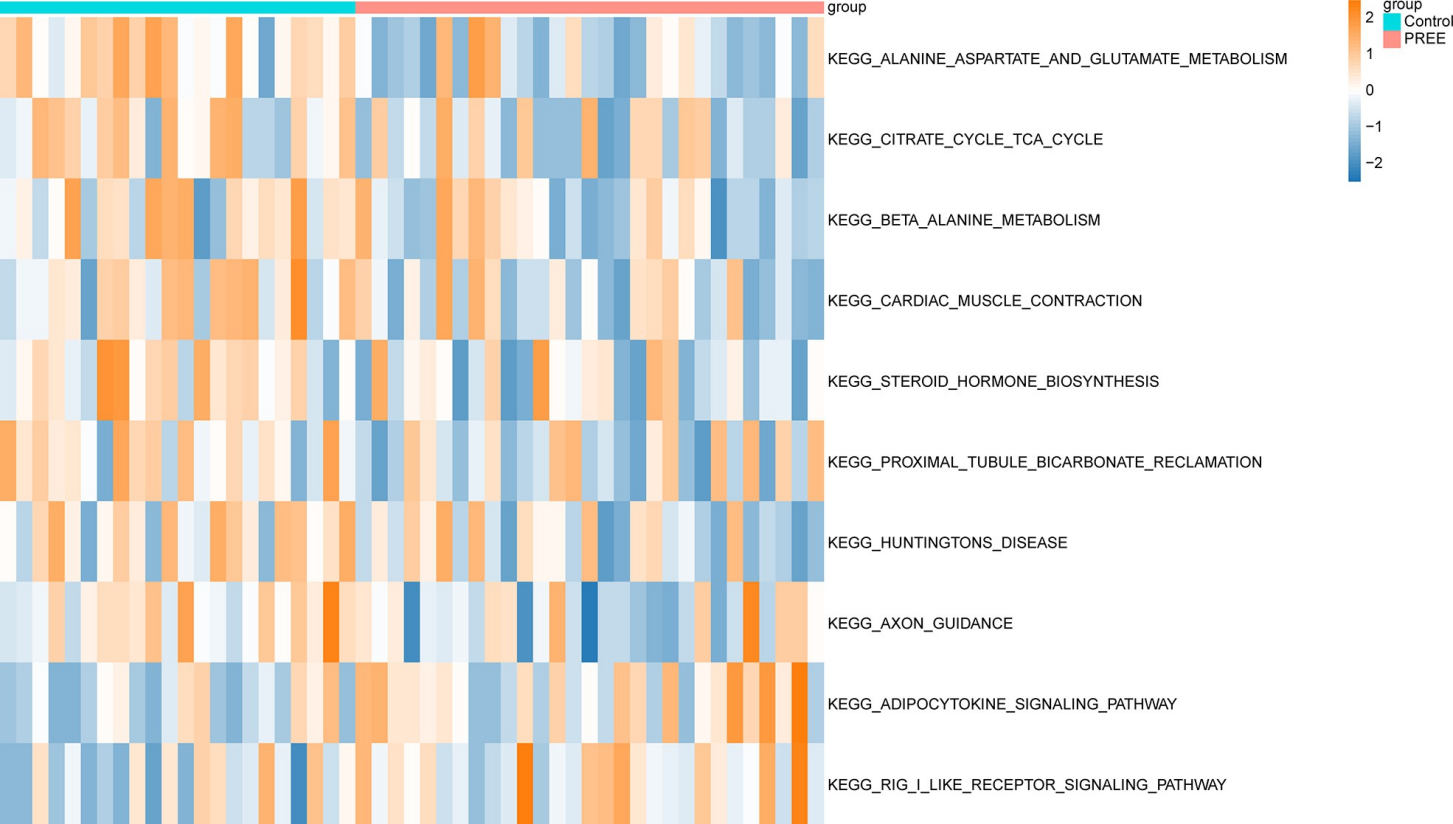
GSVA analysis.

To further explore the functional annotations between control and PE samples, GSVA analyses were performed to determine differences in pathways between the two cohorts. Multiple differentially expressed pathways were identified and then visualized using heatmaps. Compared with the control groups, the pathways associated with KEGG_BETA_ALANINE_METABOLISM and KEGG_CARDIAC_MUSCLE_CONTRACTION were markedly reduced in the PE group, whereas the expression of the KEGG_RIG_I_LIKE_RECEPTOR_SIGNALING_PATHWAY and KEGG_ADIPOCYTOKINE_SIGNALING_PATHWAY pathways was notably higher (Fig 3).

### Enrichment analyses (GO/KEGG)

**Fig 4:**
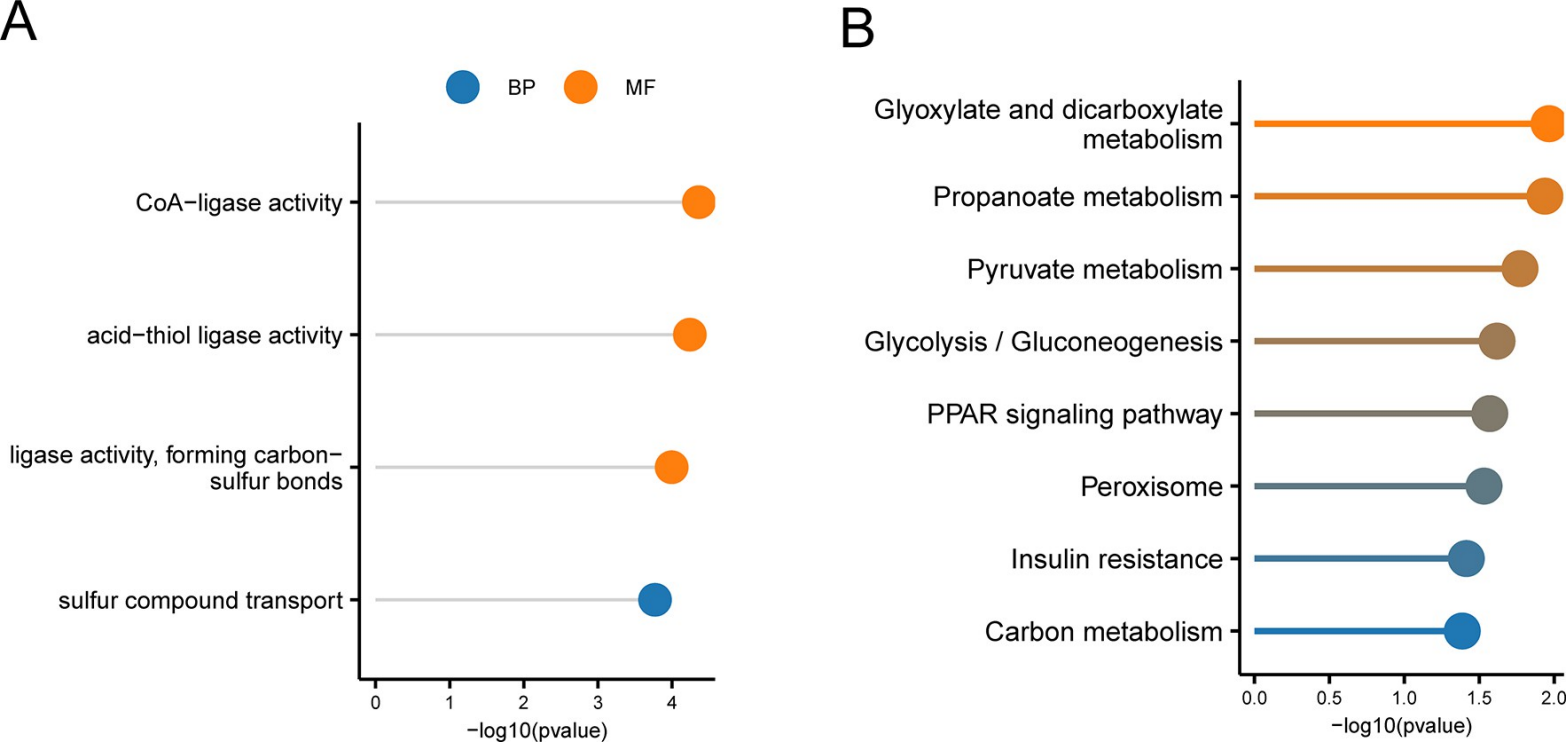
Functional enrichment of GLU-related DEGs. (A) GO enrichment. (B) KEGG enrichment.

To determine the biological activities of the GLU-related DEGs, GO and KEGG enrichment analyses were performed. The GO analysis revealed that the genes were strongly enriched in the BP category sulfur compound transport (GO:0072348) as well asacid-thiol ligase activity (GO:0016878), formation of carbon-sulfur bonds (GO:0016877), ligase activity, and CoA-ligase activity (GO:0016405) in the MF category (Fig 4A). Furthermore, the enriched KEGG pathways included glyoxylate and dicarboxylate metabolism (hsa00630), propanoate metabolism (hsa00640), and pyruvate metabolism (hsa00620) (Fig 4B).

### Validation of the hub genes

**Fig 5:**
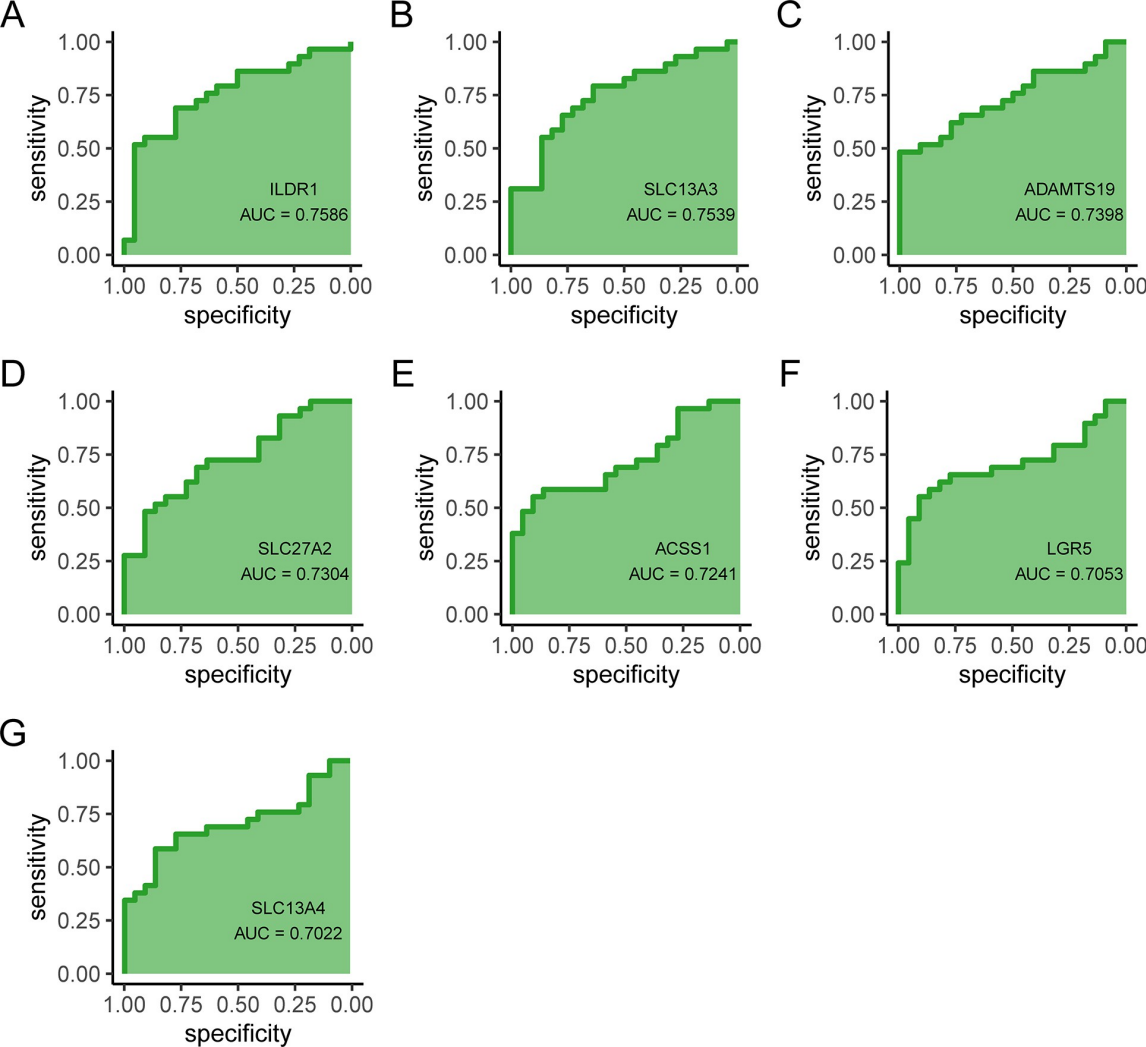
ROC curves of the hub genes. (A) ILDR1, (B) SLC13A3, (C) ADAMTS19, (D) SLC27A2, (E) ACSS1, (F) LGR5, and (G) SLC13A4.

The ROC analysis was utilized to further confirm the diagnostic value of the hub genes. The results showed that ILDR1 (AUC = 0.759), SLC13A3 (AUC = 0.754), ADAMTS19 (AUC = 0.74), SLC27A2 (AUC = 0.73), ACSS1 (AUC = 0.724), LGR5 (AUC = 0.705), SLC13A4 (AUC = 0.702) had similar AUC values (Fig 5A–5G), suggesting that the selected hub genes had the ability to discriminate between the samples and could thus be used as potential biomarkers for PE.

### Assessment of interactions between proteins

**Fig 6:**
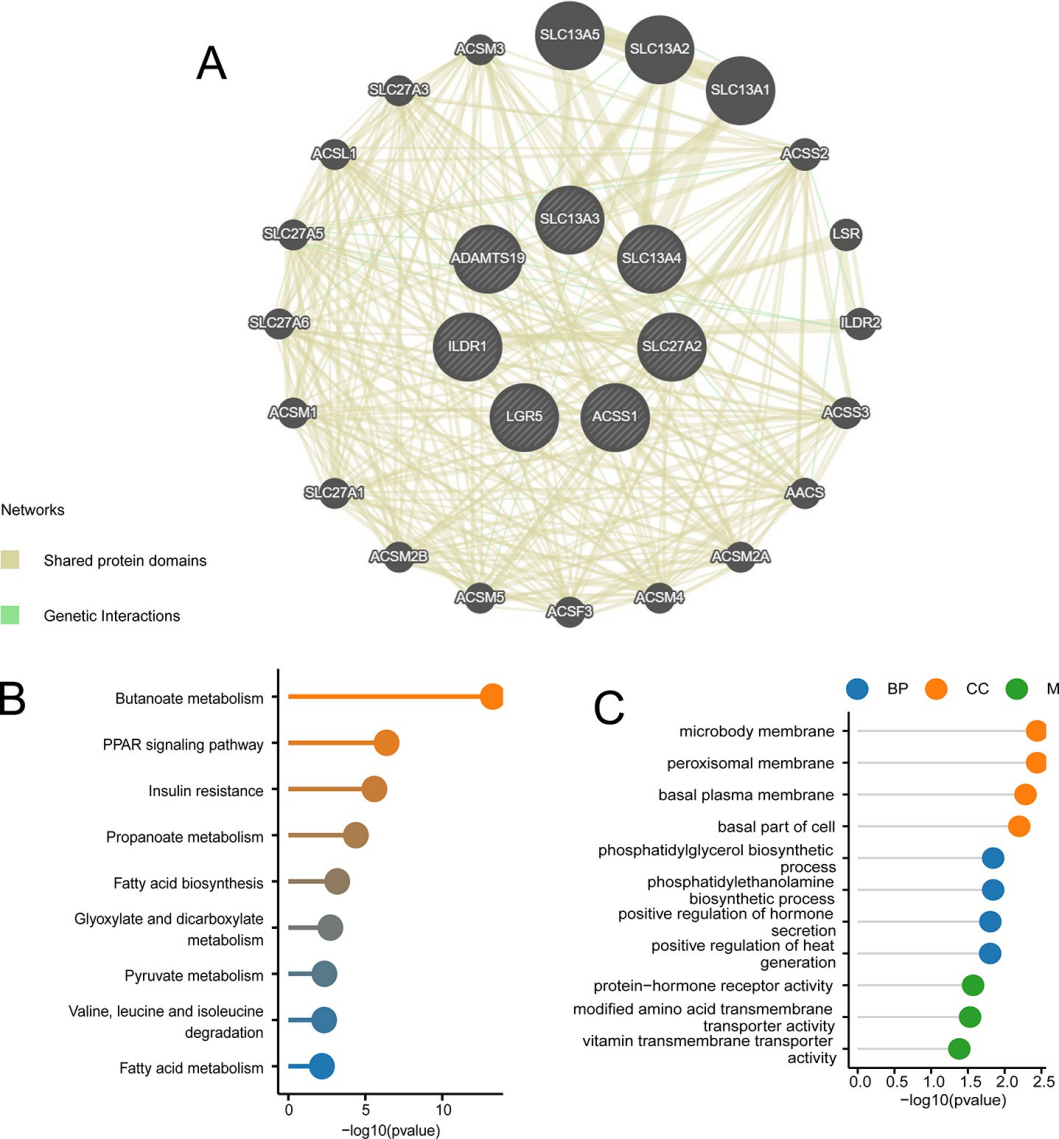
Interactions between hub genes. (A) Gene co-expression network characterization. (B) KEGG and (C) GO analyses of co-expressed genes.

The GeneMANIA database was used to generate a PPI network of the proteins encoded by the hub genes, identifying seven genes (Fig 6A). Furthermore, to elucidate the role of these genes, KEGG and GO were used to analyze enrichment of 27 genes, including 7 hub genes and 20 related genes. The KEGG analysis showed that the most significantly enriched pathways included those associated with butanoate metabolism (hsa00650), glyoxylate and dicarboxylate metabolism (hsa00630), the PPAR signaling pathway (hsa03320), insulin resistance (hsa04931), propanoate (hsa00640), fatty acid biosynthesis (hsa00061), pyruvate metabolism (hsa00620), valine, leucine, and isoleucine degradation (hsa00280), and fatty acid metabolism (hsa01212) (Fig 6B). The GO data indicated the strong enrichment of these genes in the biological processes of phosphatidylethanolamine biosynthetic process (GO:0006646), positive regulation of hormone secretion (GO:0046887), and heat generation (GO:0031652). In the CC category, enrichment was observed in peroxisomal membrane (GO:0005778), basal plasma membrane (GO:0009925), phosphatidylglycerol biosynthetic process (GO:0006655), microbody membrane (GO:0031903), and basal part of the cell (GO:0045178) and in the MF category, vitamin transmembrane transporter activity (GO:0090482), protein-hormone receptor activity (GO:0016500), and modified amino acid transmembrane transporter activity (GO:0072349) (MF) (Fig 6C).

### Validation of the gene set

**Fig 7:**
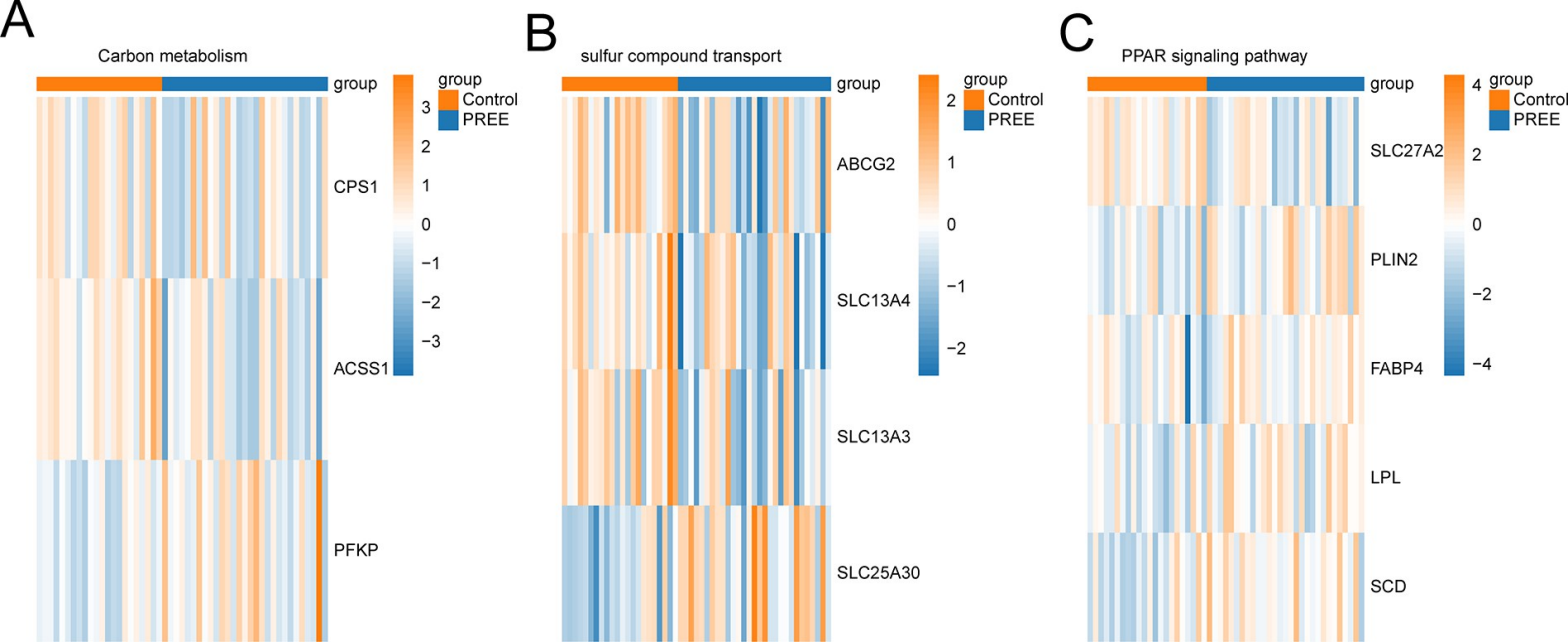
Heatmap of key genes in the gene set. (A) Carbon metabolism, (B) sulfur compound transport, and (C) PPAR signaling in PE and control samples.

The enrichment of the gene sets carbon metabolism, sulfur compound transport, and PPAR signaling pathway was validated by measurement of the expression levels of the key genes. This showed that the expression of most key genes was reduced in the PE cohort (Fig 7A–7C).

### Immune cell infiltration

**Fig 8:**
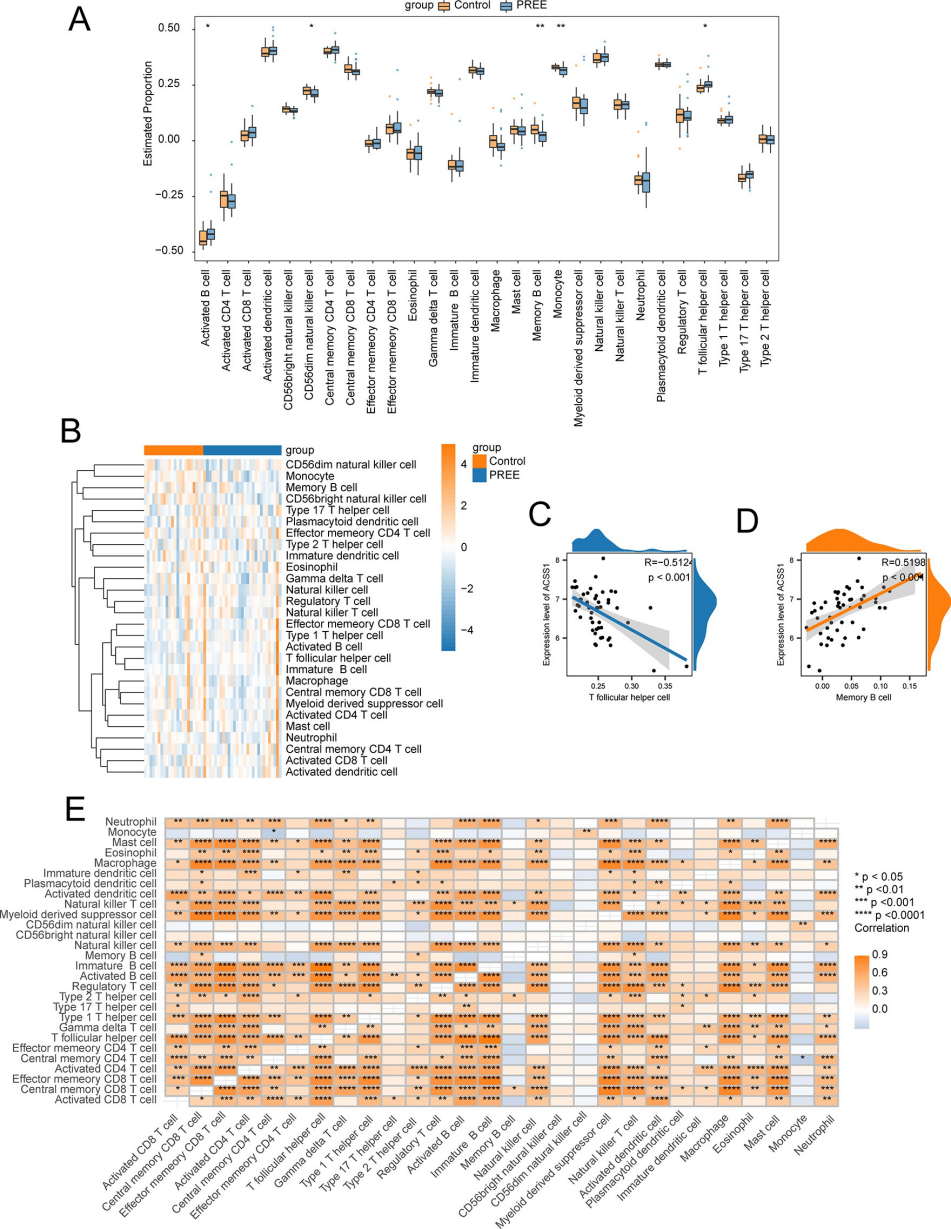
Immune infiltration analysis. (A) Estimated proportions of infiltrating immune cells in the PE and control cohorts. (B) Heatmap indicating changes in immune cell infiltration between the PE and control cohorts. (C) Correlations between the ACSS1 and TFH cells. (D) Correlations between ACSS1 and memory B cells. (E) Correlations between immune cells. Asterisks represented p-values: *p < 0.*05*, **p < 0.*01*, ***p < 0.*001*, ****p < 0.*0001*.

The infiltration of immune cells plays a critical role in PE pathogenesis. Therefore, the associations of control and PE samples with immune cell infiltration were investigated. The PE group indicated a substantially increased infiltration of two types of immune cells, namely, activated B cells and T follicular helper (TFH) cells compared with the control group (Fig 8A). As shown in Fig 8B, the overall infiltration levels of immune cells varied substantially between the PE and control groups. The infiltration of five of the overall 28 immune cell types differed significantly between the two cohorts (*p < 0*.*05*) (Fig 8B).

Furthermore, associations between the individual hub genes and immune cell infiltration were also assessed (Fig 8C and 8D). It should be noted that, ACSS1 was significantly associated with TFH cells (R = -0.512, *p < 0*.*001*) (Fig 8C), and memory B cells (R = 0.52, *p < 0*.*001*) (Fig 8D).

Subsequently, correlations between the various type of infiltrating immune cells were estimated. The majority of the immune cells were positively correlated with one another (Fig 8E).

### Signaling pathways associated with signature genes

**Fig 9:**
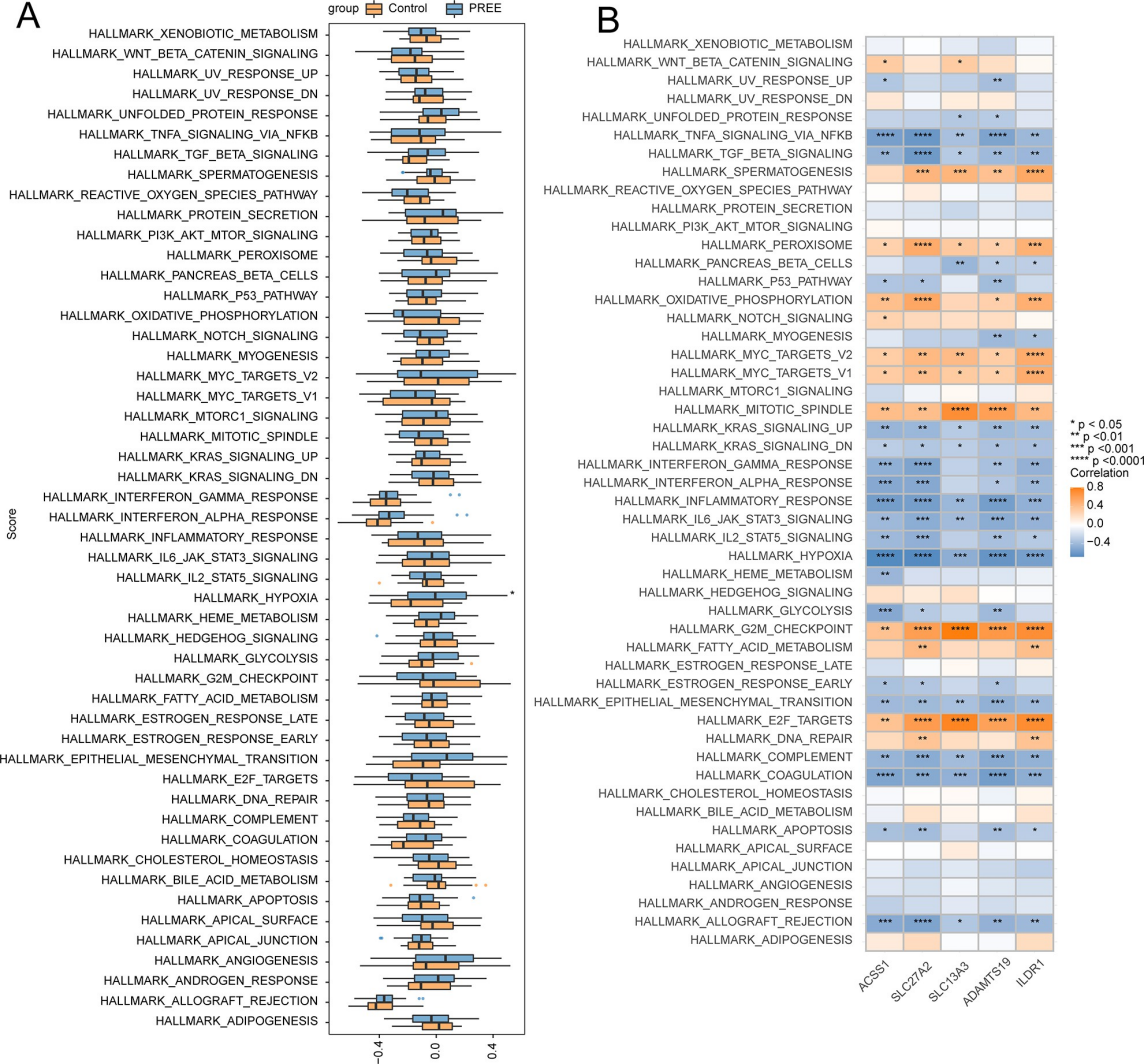
Hub genes correlated with 50 HALLMARK pathways. (A) Comparison of the 50 HALLMARK signaling pathways between the PE and control cohorts. (B) Correlation of the 50 HALLMARK signaling pathways with the target genes. *p < 0.*05*, **p < 0.*01*, ***p < 0.*001*, ****p < 0.*0001*.

With the help of GSVA, the variability between PE and controls was assessed in 50 HALLMARK signaling pathways. In PE patients, one HALLMARK signaling pathway was found to be significantly up-regulated, namely, HALLMARK_HYPOXIA, and no pathways were down-regulated (Fig 9A).

Furthermore, the correlations between 50 HALLMARK signaling pathways and the top five differentially expressed hub genes were elucidated. This showed that ACSS1 was associated with various pathways, including HALLMARK_E2F_TARGETS (Fig 9B).

### Construction and functional annotation of crosstalk between RBPs and hub mRNAs

**Fig 10:**
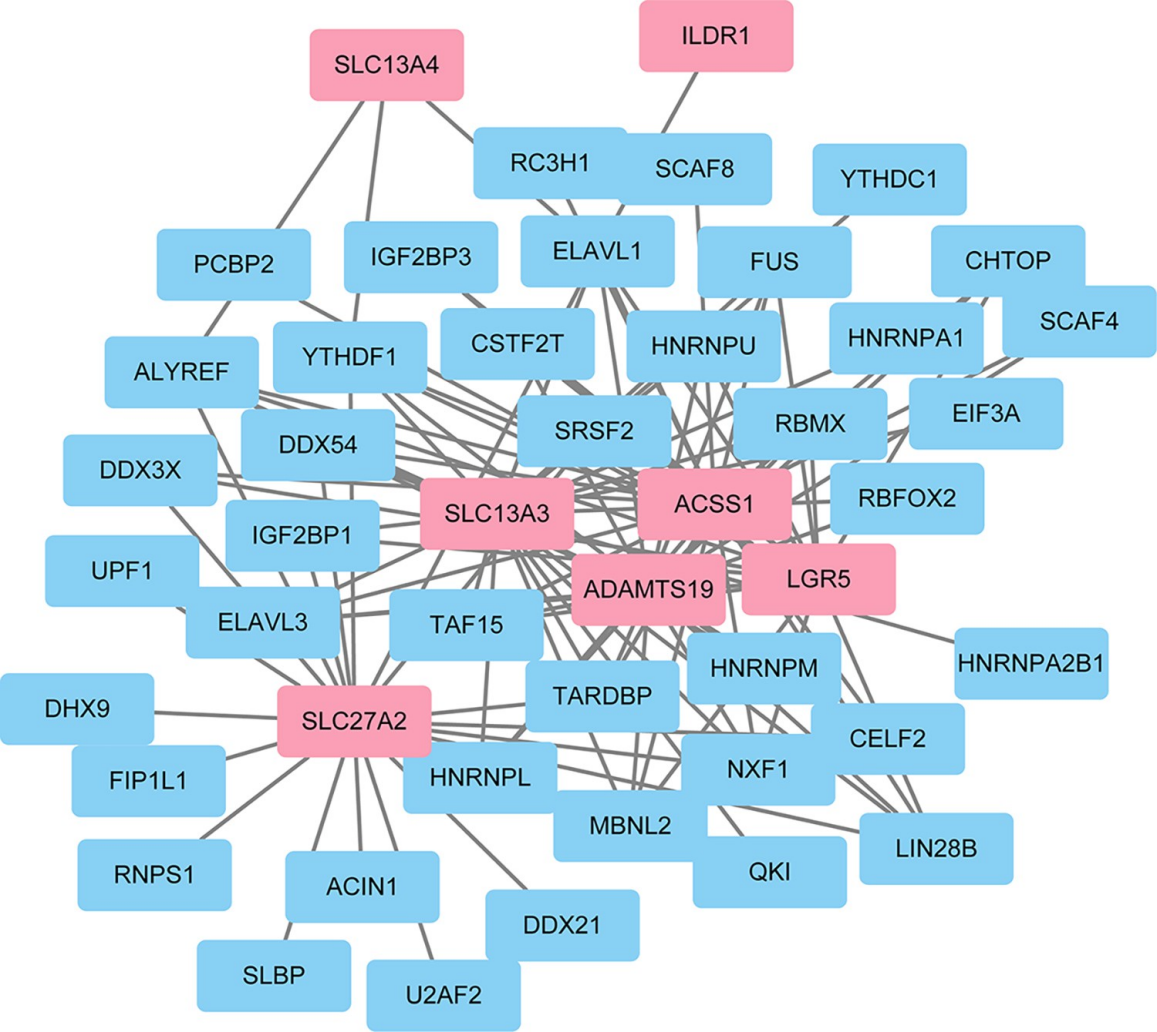
The RBP-mRNA regulatory network. Pink = mRNAs and blue = RBPs.

As RBPs interact with mRNAs, 7 hub mRNAs were selected using StarBase, and 7 mRNA/RBP pairs were identified and imported. An RBP-mRNA network was established on the basis of the link between target genes acquired from the online dataset. This network comprised 7 mRNAs, 40 RBPs, 47 nodes, and 105 edges. Fig 10 illustrates the network.

### Verification of hub genes

**Fig 11:**
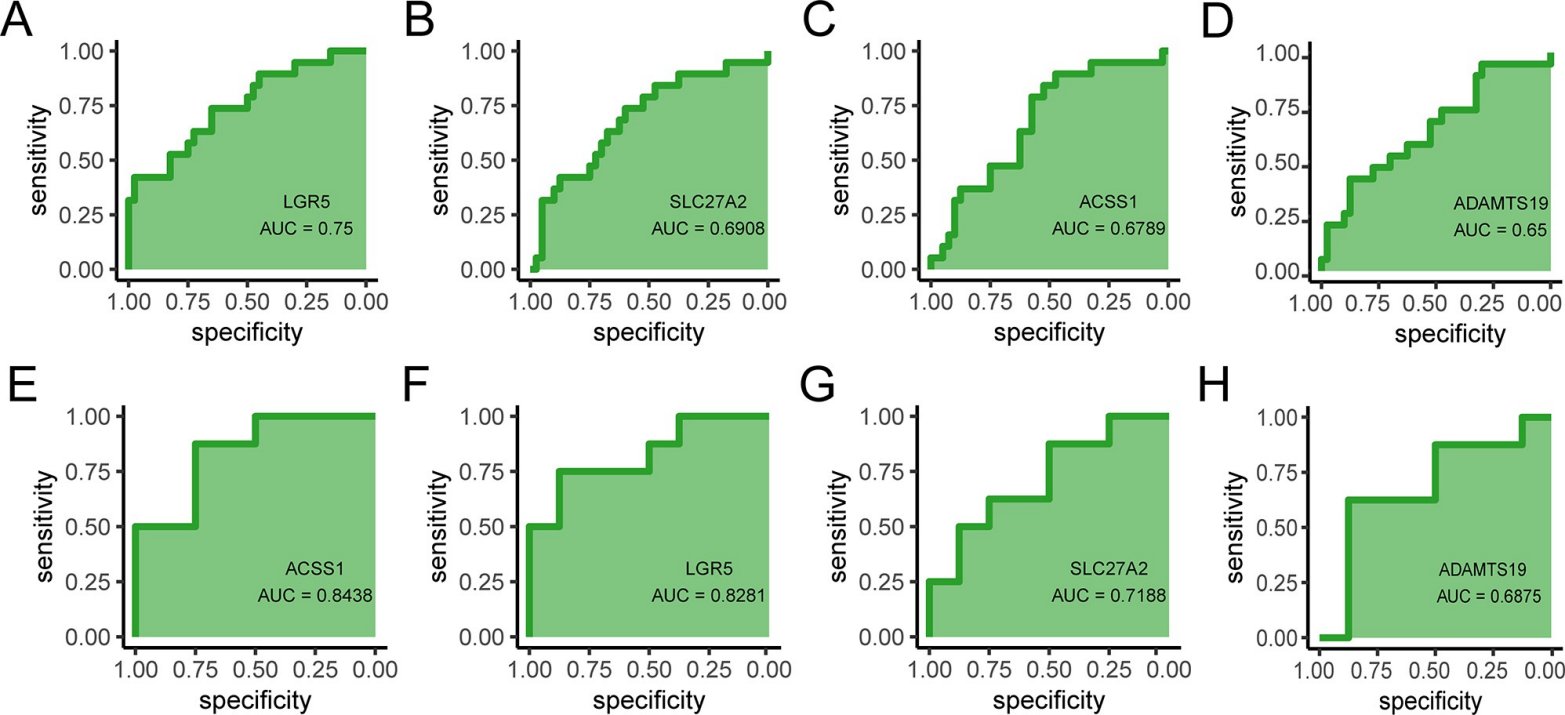
Verification of the hub genes in datasets. LGR5. (A), SLC27A2 (B), ACSS1 (C), ADAMTS19 (D) in the GSE35574 dataset and ACSS1 (E), LGR5 (F), SLC27A2 (G), ADAMTS19 (H) in the GSE24129 dataset.

The value of the hub genes as potential biomarkers was assessed using ROC curve analysis of two independent GEO datasets, GSE24129 and GSE35574, to validate their diagnostic performance in PE. The AUC values that in the dataset GSE35574 (Fig 11A–11D), LGR5 (AUC = 0.75), SLC27A2 (AUC = 0.691), ACSS1 (AUC = 0.679), ADAMTS19 (AUC = 0.65), and in the dataset GSE24129 (Fig 11E–11H), ACSS1 (AUC = 0.844), LGR5 (AUC = 0.828), SLC27A2 (AUC = 0.719), ADAMTS19 (AUC = 0.688), indicated that that the hub genes ADAMTS19, SLC27A2, ACSS1, and LGR5 were potential biomarkers of PE.

### Verification of hub genes in clinical samples

**Fig 12:**
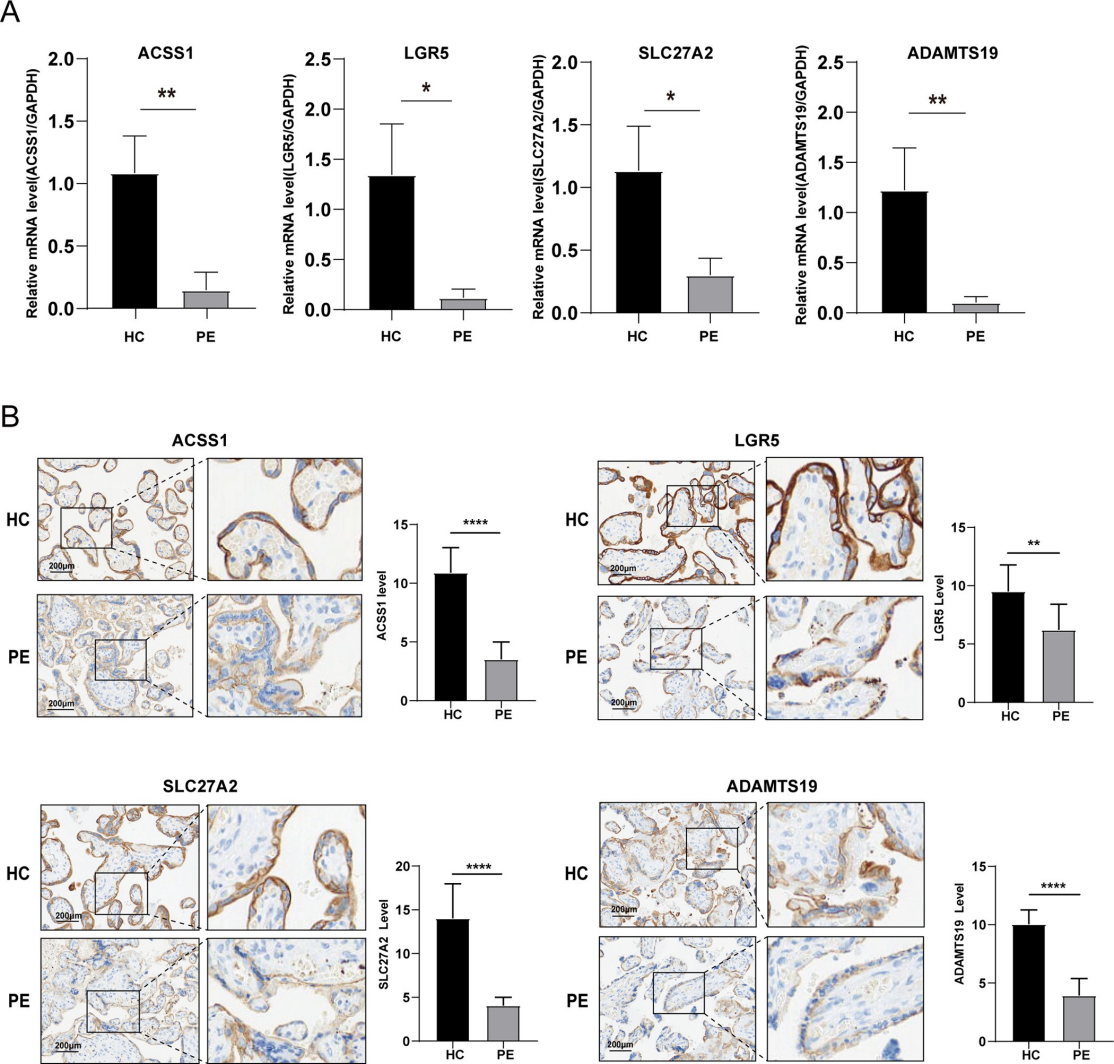
Validating hub gene expression in clinical samples. (A) Relative mRNA expression of hub genes in the placental tissue of HC and PE. (B) Immunohistochemical analyses of paraffin-embedded placental tissue of HC and PE.****p < 0.*0001*, **p < 0.*01*, *p < 0.*05*.

The expression levels of the hub genes in the placental tissue of patients in the PE and HC groups were determined by quantitative RT-PCR and Immunohistochemical (IHC). Thus indicated that the expression of ADAMTS19, SLC27A2, ACSS 1, and LGR 5 were reduced in PE patients relative to the control group (Fig 12A and 12B), consistent with the bioinformatics analysis. The clinical data of HC pregnant women and PE patients are shown in S1 Table.

## Discussion

Preeclampsia is a placenta-associated disorder that causes serious complications to both the mother and child, including eclampsia, cerebrovascular accidents, fetal growth restriction, and placental abruption. Due to the absence of early diagnostic markers, women with PE often miss important treatment opportunities and times, resulting in poor prognosis [20,21]. Furthermore, the literature indicates that immune cell infiltration is closely linked with the incidence of PE. A serum metabolomic analysis found clear differences in GLU metabolism between women with normal pregnancies and those with PE [22]. Therefore, identifying specific diagnostic indices and elucidating immune cell infiltration in PE could profoundly improve its prognosis. Bioinformatics is a powerful tool used to identify molecular indices. The present study identified diagnostic indices for PE and explored the role of infiltrating immune cells in PE.

In addition to maintaining the cellular redox state and antioxidant function, glutaminolysis is associated with the biosynthesis of many essential molecules by providing a carbon source for synthetic lipid compounds. Fatty acids are an important substrate for energy supply; therefore, sufficient fatty acid metabolism is essential during pregnancy, and reduced expression of fatty acid metabolic genes in the placenta has been associated with PE [23]. Among the 7 hub genes (ILDR1, SLC13A3, ADAMTS19, SLC27A2, ACSS1, LGR5, and SLC13A4) identified in this study, SLC13A3 is a transporter of citrate, and tricarboxylate, ACSS 1 is a mitochondrial matrix enzyme that promotes acetyl-CoA production, a precursor for fatty acid synthesis [24,25], while SLC27A2 transports fatty acids in oxidative stress and damage to endothelial cells [26]. In addition, citrate is also predictive of PE [27]. Immunoglobulin-like domain-containing receptor 1 (ILDR 1) is a member of the lipoprotein residual receptor family and it has been found that the loss of ILDR 1 results in the inhibition of glucose metabolism in mice [28]. SLC13A4 is a sodium sulfate transporter protein that is mainly expressed in the placenta, and its reduced activity results in defective placental development [29]. ADAMTS19 is a member of the metalloproteinase family that can cleave the components of the extracellular matrix or interact with modulatory factors, affecting cell migration, adhesion, growth, and angiogenesis [30]; an early absence of placental trophoblast invasion is one of the mechanisms associated with PE. Moreover, it has been suggested that ADAMTS19 also affects the biological activity of trophoblast cells; however, further experimental research is required.

The GO and KEGG enrichment analyses identified pathways associated with sulfur compound transport, PPAR signaling, and energy metabolism. Sulfur compounds include sulfate and sulfur-containing amino acids, which are obligate nutrients for healthy development and growth and can be acquired from intracellular sulfur amino acids metabolism and the diet [31]. In PE, the levels of sulfite, sulfur amino acid ergotine, and hydrogen sulfide are markedly reduced [32–34]. Immune functions and metabolism are the core mechanisms used by the body to maintain tissue homeostasis [35]. The metabolism of immune cells is closely related to their phenotype and function [36]. The phenotypic and functional abnormalities of decidual immune cells, such as macrophages and NK cells, can cause dysfunctional trophoblast invasion and defective spiral artery reconstruction, which is the main pathogenesis of PE [37]. PPARs belong to the nuclear hormone receptor family and are implicated in lipid metabolism, immune function, cell growth, and differentiation. Furthermore, free fatty acids utilize PPARs to influence macrophage function and differentiation [38]. Lipids can participate indirectly as substrates or directly as disease mediators in PE etiology [39]. Lipid peroxides can activate endothelial cells and reinforce the pathology of diabetes mellitus and essential hypertension, which are significant risk factors for PE [40]. PPARs can also directly upregulate the expression of fatty acid-binding protein (FABP) and promote fatty acid uptake and accumulation in trophoblast cells, thus inhibiting their invasion ability [41].

GSVA provides valuable data on numerous genes with a relatively smaller fold changes. Here, GSEA analysis was conducted on the gene profiles of the datasets, which revealed multiple gene sets that were markedly enriched in the PE cohort. Among them, the expression of an adipocytokine signaling pathway was significantly increased; this included the synthesis and secretion of many hormones and cytokines by adipocytes to regulate energy metabolism, inflammation, the immune response, cell proliferation, and other physiological functions by binding to cell-surface receptors. The association of various adipokines with insulin resistance and atherosclerosis highlights its importance in metabolic diseases. There are many studies on adipokines and PE. Furthermore, C1q-TNF-related protein 9 (CTRP 9) can affect trophoblast cell proliferation and migration and cause PE [42]. Moreover, Apelin improves endothelial dysfunction to alleviate acute liver and kidney injury in PE [43]. Additionally, a birth credit analysis suggested that adipokine leptin may be a biomarker of PE [44].

To further elucidate the activities of infiltrating immune cells in PE, ssGSEA was employed, which indicated that enhanced infiltration of activated B and TFH cells and reduced infiltration of CD56 bright NK cells, monocytes, and memory B cells might be linked with the incidence of PE. The correlation of 7 hub genes with infiltrating immune cells showed significant negative associations with activated B and TFH cells. Both T and B cells participate in humoral immunity, and increased levels of pro-inflammatory T cells can lead to oxidative stress and cause chronic inflammation characterized by increased production of pro-inflammatory factors and autoantibodies [45]. Women with PE often show superficial chronic inflammatory trophoblast invasion, and the study found that B cell levels were markedly elevated in the peripheral blood of PE patients [46,47]; B cells can induce the production of pro-inflammatory T cells [48]. TFH cells can secrete cytokines to stimulate B cell proliferation. Therefore, it is suggested that the reduced expression of these 7 hub genes might increase the production of activated B and TFH cells and participate in the occurrence and development of PE. These hypotheses require more research to determine the complex communication between genes and immune cells.However, we recognize the limitations in immediate clinical application and the importance of exploring peripheral blood-based biomarkers for practical diagnostic purposes. We will consider expanding our analysis to include peripheral blood samples in future research to enhance clinical relevance.

## Conclusion

In summary, this study identified infiltrating immune cells, WGCNA modules, DEGs, enriched pathways, and hub genes that might be associated with PE pathogenesis. These data furnish novel evidence for understanding PE pathogenesis and lays the foundation for future studies aimed at developing clinically relevant diagnostic and therapeutic strategies.

## Supporting information

S1 Fig Workflow. (TIF)

S1 Table Clinical characteristics of preeclamptic and health control pregnancies. (XLSX)

S1 Dataset (ZIP)
