# Supplementary figures and images for: Identification of hub glutamine metabolism-associated genes and immune characteristics in pre-eclampsia
Source: PLoS One. 2024 May 8;19(5):e0303471. doi: 10.1371/journal.pone.0303471 (PMC11078374; doi:10.1371/journal.pone.0303471)

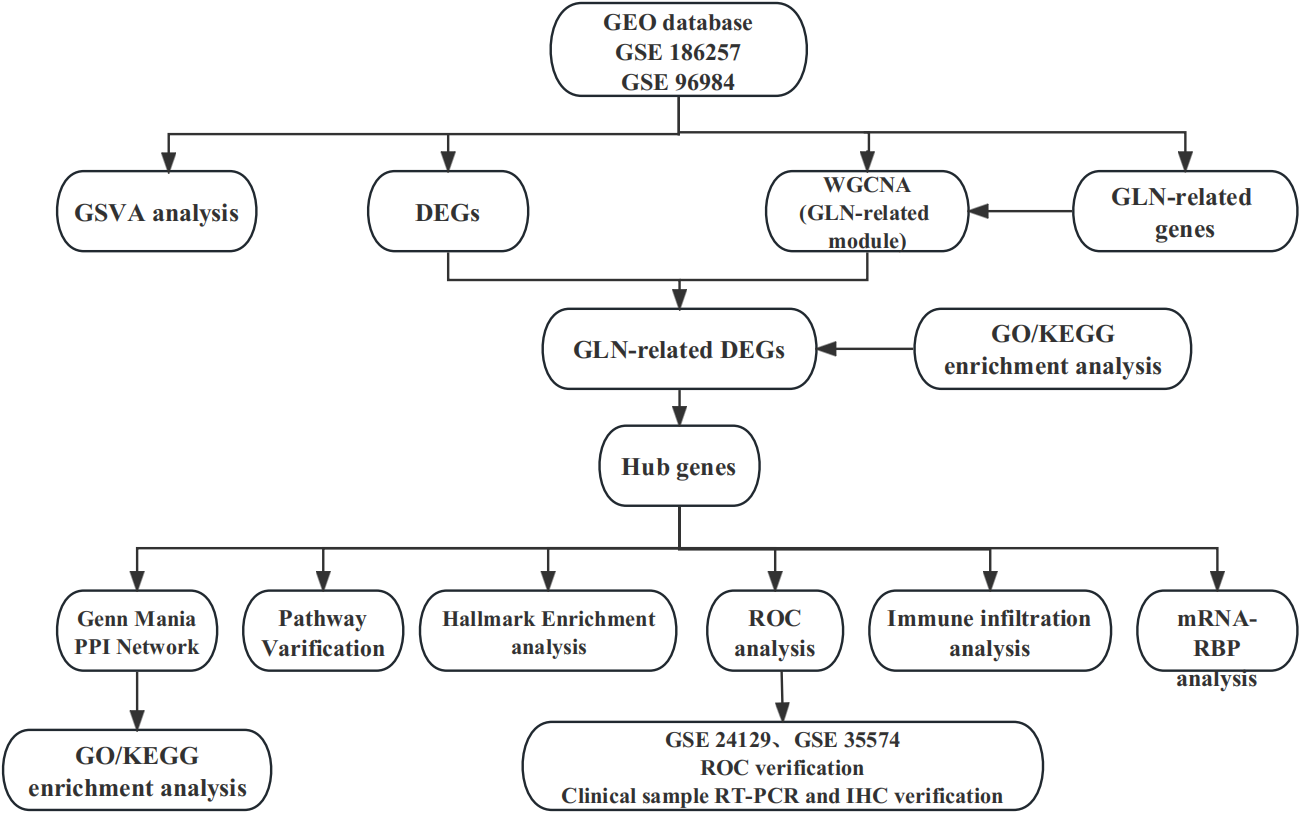

Supplement: S1 Fig — (TIF) [file pone.0303471.s001.tif]
